# Expression of a manganese peroxidase isozyme 2 transgene in the ethanologenic white rot fungus *Phlebia* sp. strain MG-60

**DOI:** 10.1186/2193-1801-3-699

**Published:** 2014-11-27

**Authors:** Yumi Yamasaki, Megumi Yamaguchi, Kenji Yamagishi, Hirofumi Hirai, Ryuichiro Kondo, Ichiro Kamei, Sadatoshi Meguro

**Affiliations:** Center of Community Organization, University of Miyazaki, 1-1 Gakuenkibanadai-nishi, Miyazaki, 889-2192 Japan; Department of Forest and Environmental Sciences, Faculty of Agriculture, University of Miyazaki, 1-1 Gakuenkibanadai-nishi, Miyazaki, 889-2192 Japan; Planning and Promotion Section, NARO Tohoku Agricultural Research Center for Tohoku Region, Morioka, 020-0198 Japan; Department of Applied Biological Chemistry, Faculty of Agriculture, Shizuoka University, Shizuoka, 422-8529 Japan; Department of Agro-Environmental Sciences, Faculty of Agriculture, Kyushu University, Fukuoka, 812-8581 Japan

**Keywords:** Lignin, Manganese peroxidase, *Phlebia* sp. MG-60, White rot fungus

## Abstract

**Background:**

The white-rot fungus *Phlebia* sp. strain MG-60 was proposed as a candidate for integrated fungal fermentation process (IFFP), which unifies aerobic delignification and semi-aerobic consolidated biological processing by a single microorganism based on its ability to efficiently degrade lignin and ferment the sugars from cellulose. To improve IFFP, the development of a molecular breeding method for strain MG-60 is necessary. The purpose of this study is to establish the transformation method for the strain MG-60 and to obtain the over-expressing transformants of lignin-degrading enzyme, manganese peroxidase.

**Findings:**

In the present study, the expression vector regulated by *Phlebia brevispora* glyceraldehyde-3-phosphate dehydrogenase promoter and terminator was constructed. A polyethylene glycol transformation method for the ethanol-fermenting white-rot fungus *Phlebia* sp. MG-60 was established with high transformation efficiency, and the manganese peroxidase isozyme 2 gene (MG*mnp2*) transformants were obtained, showing higher MnP activity than control transformants. MG*mnp2* transformants showed higher selective lignin degradation on *Quercus* wood powder.

**Conclusions:**

This first report of MG-60 transformation provides a useful methodology for widely accessible to interested researches. These results indicate the possibility of metabolic engineering of strain MG-60 for improving IFFP.

**Electronic supplementary material:**

The online version of this article (doi:10.1186/2193-1801-3-699) contains supplementary material, which is available to authorized users.

## Introduction

The white-rot fungus *Phlebia* sp. strain MG-60 was proposed as a candidate for integrated fungal fermentation process (IFFP), which unifies aerobic delignification and semi-aerobic consolidated biological processing by a single microorganism based on its ability to efficiently degrade lignin and ferment the sugars from cellulose (Kamei et al. 2012a, [b]). To improve this environmentally friendly and unique process for biorefinery of lignocellulose, the development of a molecular breeding method for strain MG-60 is necessary. Manganese peroxidase (MnP) is considered a key enzyme in the lignin degrading basidiomycetous fungi, which catalyzes the oxidation of Mn^2+^ to Mn^3+^ along with an H_2_O_2_ generating system (Gold and Alic 1993; Martínez 2002). Three MnP encoding genes (MG*mnp1*, MG*mnp2* and MG*mnp3*) were cloned from the strain MG-60 (Kamei et al. 2008). It is expected that the overexpression of MnP genes improves delignification ability of the strain. In this study, an expression vector was constructed, and transformation efficiency was evaluated. Then, MG*mnp2* expressing transformants were generated, and the MnP activity on the transformants was determined. Additionally, the ability of the transformants to degrade lignin was analyzed.

## Materials and methods

### Construction of expression vectors

*Phlebia* sp. strain MG-60 TUFC40001 (Fungus/Mushroom Resource and Research Center, Tottori, Japan) was maintained on potato dextrose agar (PDA) plates. Additional file 1: Figure S1 shows the construction procedure for the expression plasmids p*PbGPD* (glyceraldehyde-3-phosphate dehydrogenase)*-HPT*, p*PbGPD-EGFP* and p*PbGPD-*MG*mnp2*. Additional file 1: Table S1 lists the primers (Life Technologies, Carlsbad, CA) used. The *PbGPD* gene was obtained from *Phlebia brevispora* HHB-7030 genomic DNA (protein ID: 29450) by PCR amplification with the primers *PbGPD*-F1 and *PbGPD*-R1, and the amplified fragment was ligated into the T-Vector pMD20 (TAKARA BIO INC, Shiga, Japan) (steps 1 and 2). The added *Asc*I restriction enzyme sites, included in the *PbGPD* promoter and *PbGPD* terminator, were obtained using the primers *PbGPD-Asc-*F1 and *PbGPD-Asc-*R1 (step 3). *HPT*, *EGFP* and MG*mnp2* genes containing the added *Asc*I restriction enzyme site were amplified with PCR by using primers *gHPT-Asc-*F1, *gHPT-Asc-*R1, *gEGFP-Asc-*F1, *gEGFP-Asc-*R1, *g*MG*mnp2-Asc-*F1 and *g*MG*mnp2-Asc-*R1, which were designed based on the *Escherichia coli* hygromycin B phosphotransferase gene (accession number: K01193), the *Aequorea victoria* EGFP gene (accession number: U55761) and the *Phlebia* sp. MG-60 MG*mnp2* gDNA sequence data (accession number: AB971352), respectively. The amplified *HPT, EGFP* and MG*mnp2* genes were ligated into the expression plasmid after digestion by *Asc*I (New England Biolabs Japan Inc., Tokyo, Japan) according to DNA Ligation Kit Mighty Mix (TAKARA BIO INC, Shiga, Japan) instructions, and then transformed into competent *E. coli* JM 109 (TAKARA BIO INC, Shiga, Japan) for amplification.

### Transformation

Protoplast isolation and polyethylene glycol (PEG)-mediated co-transformation assays with MG-60 were performed in a manner similar to the method reported with *Phanerochaete sordida* YK-624 previously (Yamagishi et al. 2007). Briefly, MG-60 was pre-cultured in 100 mL CYM (yeast extract 2 g/L, polypeptone 2 g/L, glucose 20 g/L, MgSO_4_-7H_2_O 0.5 g/L, KH_2_PO_4_ 0.46 g/L, K_2_HPO_4_ 1 g/L, vitamin B_1_ 1 mg/L, thiabendazole 20 μg/L, pH 6.0) medium for 3 d without shaking. After pre-culture, the mycelia were homogenized for 10 sec and sub-cultured for 3 d in CYM medium. The mycelia were harvested by filtration and treated with 0.5 M MgSO_4_ buffer (0.5 M MgSO_4_-7H_2_O, 20 mM maleic acid) containing 2.5% (w/v) Cellulase Onozuka (Yakult, Tokyo, Japan) and 2.5% (w/v) Lysing Enzymes from *Trichoderma* (Sigma-Aldrich, MO, USA) at 30°C on a shaker NTS-4000B (TOKYO RIKAKIKAI CO, LTD, Tokyo, Japan) at 60 rpm for 4 h. The mycelial suspension was overlaid with 1.0 M SorbOsm (1.0 M sorbitol, 10 mM MES, pH 6.3) and centrifuged at 1,500 *g* in a TOMY LC-122 centrifuge (TOMY SEIKO CO., LTD, Tokyo, Japan) for 20 min. Protoplasts that accumulated at the interface between the two liquid phases were collected, equaling approximately 7.5 × 10^6^ protoplasts per 1 g of wet mycelial weight. Then, 1.5 × 10^6^ protoplasts in 500 μL of 1.0 M SorbOsm were treated with 300 μL of DNA solution containing 20 μg plasmid in 1 M sorbitol and 0.04 M CaCl_2._ The tube was placed on ice for 30 min. After treatment, 800 μL of 50% (w/v) PEG4000 and then 75 mL regeneration medium were added to the tube. The entire contents were poured onto 50 petri dishes (2.0 × 10^4^ cells/mL), and incubated at 30°C. Transformants with hygromycin resistance were selected by treatment with 15 μg/mL hygromycin B (Life Technologies, Carlsbad, CA). *EGFP* and MG*mnp2* transformants were selected by genomic PCR amplification with the primers *PbGPD*-*prom*-F1, *gEGFP-Asc-*R1 and *g*MG*mnp2-Asc-*R1.

### MnP activity

The transformants with high MnP activities were selected by measuring the activity. Wild type MG-60 (Wt), *HPT*-expressing transformants (HPT) and MG*mnp2*-expression candidates (M1 to M14) were cultured in 8 mL Kirk’s high-nitrogen medium (Kirk HN) (Tien and Kirk 1988) at pH 4.5 in test tubes on a reciprocal shaker MMS-3010 shaker (TOKYO RIKAKIKAI CO, LTD, Tokyo, Japan) at 150 rpm in the dark at 28°C for 3 or 6 d. After incubation, the whole culture was separated into mycelium and extracellular fluid by centrifugation at 12,000 *g* in a centrifuge 5430R (Eppendorf AG, Hamburg, Germany) for 10 min. MnP activity was measured as described by Martínez et al. ( 1996).

### Treatment of wood powder

The transformants selected above were pre-cultured on PDA plates at 28°C. The solid medium of *Quercus serrata* Thun*b.*wood powder was adjusted to 80% moisture content using distilled water and sterilized at 121°C for 15 min. One mycelial disk (5 mm in diameter), punched from colony edges on PDA, were inoculated to the surface of the prepared medium, and then incubated at 28°C for 20 d. The test was carried out independently using three flasks per each strain. After incubation, the samples were dried in the oven DX-58 (Yamato Scientific Co., Ltd., Tokyo, Japan) at 105°C, and the dry weight was measured. The Klason lignin content of the samples was determined according to the standard analytical laboratory procedure (Sluiter et al. 2011). The selective ability of the transformants to degrade lignin was calculated as the ratio of lignin mass loss to the mass of wood powder (the *L/W* ratio) (Suhara et al. 2012). To measure the MnP activity of the transformants, samples were extracted with 20 mL of 71 mM malonic acid buffer on ice for 1 h with stirring every 10 min. Data were analyzed by Dunnett’s test to evaluate the significance of differences, and *p* <0.05 was regarded as statistically significant.

## Results and discussions

As preliminary experiments, the protoplast regeneration and transformation efficiency of strain MG-60 was measured. The regeneration value on the plate without hygromycin B was 86.7% (Table 1). The presence of hygromycin B completely inhibited the viability of MG-60. The selective marker p*PbGPD-HPT* was introduced into the protoplasts of MG-60, and the growth of *HPT* transformants was observed. On average, approximately 10 colonies per petri dish with hygromycin B showed growth after 7 d of culturing. The transformation efficiency (Table 1) was higher than that of previous reports with other white rot fungi (Akileswaran et al. 1993; Bartholomew et al. 2001; Yamagishi et al. 2007). These results indicate that the protoplast-PEG method using constructed expression plasmids is viable for the transformation of strain MG-60.

**Table 1:** **Transformation efficiency of** ***Phlebia*** **sp. strain MG-60 with p** ***PbGPD-HPT,*** **p** ***PbGPD-EGFP*** **and p** ***PbGPD-*** **MG** ***mnp2***

Transformed plasmid	Hygromycin B	Protoplast regeneration efficiency (%) ^a^	Co-transformation efficiency (%) ^b^
-	-	86.7	-
-	+	0.0	-
p*PbGPD-HPT*	+	-	0.042 (549 colonies)
p*PbGPD-HPT*, p*PbGPD-EGFP*	+	-	0.029 (380 colonies)
p*PbGPD-HPT*, p*PbGPD-*MG*mnp2*	+	-	0.038 (489 colonies)
Transformants		Transformation efficiency (%)	
*EGFP* transformants/*HPT* transformants		88.9 (32 clones/36 clones)	
MGm*np2* transformants/*HPT* transformants		89.1 (181 clones/203 clones)	

p*PbGPD-HPT* indicates Hygromycin-resistance gene expression plasmid, p*PbGPD-EGFP* indicate enhanced green fluorescent protein gene expression plasmid, and p*PbGPD-*MG*mnp2* indicate manganese peroxidase isozyme 2 gene expression plasmid derived from MG-60.

^a^The protoplast regeneration efficiency per protoplast: (regenerated clones/seeding protoplasts).

^b^The transformation efficiency per protoplast: (*HPT* transformants/regenerated clones).

To confirm the antibiotic resistance of the transformants and eliminate the possibility of false-positives, expression of the transgene was confirmed. p*PbGPD-EGFP* was used to co-transform strain MG-60 with p*PbGPD-HPT*, and transformants that contained both plasmids and expressed EGFP were obtained. The expression of p*PbGPD-EGFP* could be assessed by the presence or absence of EGFP fluorescence under the microscope*.* As shown in Table 1, the transformation efficiency of hygromycin-resistant transformants was 0.042%, and EGFP positive transformants accounted for nearly 90% of the *HPT* transformants. EGFP fluorescence was observed in the transformants under the microscope (Additional file 1: Figure S2). This is the first study showing that *Phlebia* sp. strain MG-60 is able to be co-transformed by the protoplast-PEG method.

**Figure 1:**
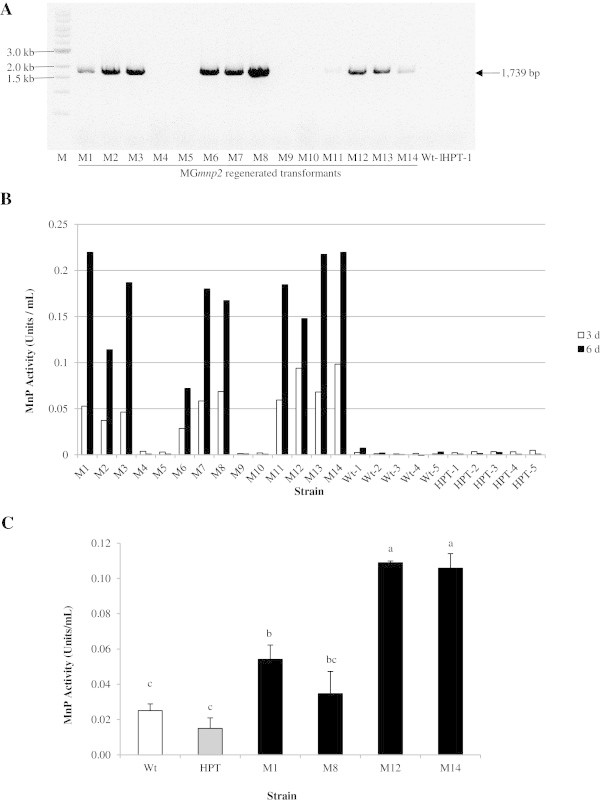
**Co-transformation of** ***Phlebia*** **sp. strain MG-60 with p** ***PbGPD*** **-MG** ***mnp*** **2 and p** ***PbGPD-HPT*** **. A**, Detection of the MG*mnp2* gene in 10 of 14 regenerated protoplasts co-transformed with p*PbGPD-HPT* and p*PbGPD*-MG*mnp*2 by PCR amplification using primers *PbGPD-prom*-F1 and *g*MG*mnp2-Asc-*R1. M indicates a 1 kb ladder size marker, Wt indicates the wild type, HPT indicates the hygromycin resistant transformant, and M1–M14 indicate MG*mnp2* regenerated transformants. **B**, MnP activity in Kirk’s high-nitrogen culture medium under aerobic condition for 3 d (white column) and 6 d (black column). One unit of MnP activity was defined as 1 μmol reaction product formed per minute. **C**, MnP activity of various strains in the extracts of *Quercus* wood powder medium under aerobic condition for 20 d. The tests, using three flasks per strain, were carried out independently. Data are means ± SE (n = 3) and values without a common superscript letter are significantly different at *p* <0.05.

*Phlebia* sp. MG-60 was able to degrade lignin and to brighten the unbleached hardwood kraft pulp extensively under hypersaline environment (Li et al. 2002). Expression of MG*mnp2* was induced under hypersaline condition (Kamei et al. 2008). Therefore, it was expected that overexpression of MG*mnp2* improve delignification ability of strain MG-60 under non-saline condition. To obtain MG*mnp2* transformants, 14 *HPT* transformants were selected, the insertion of p*PbGPD-*MG*mnp2* was confirmed, and the MnP activity produced by the each transformant was evaluated. The insertion of p*PbGPD-*MG*mnp2* was observed in 10 of the 14 *HPT* transformants (Figure 1A). As shown in Figure 1B, the 10 MG*mnp2* positive transformants had higher MnP activities than all Wt and p*PbGPD-*MG*mnp2* negative transformants in Kirk HN medium. These data indicate that expression of the MG*mnp2* transgene was translated successfully into active MnP2.

To evaluate the effects of the expression of the MG*mnp2* transgene on wood degradation traits, treatment of *Quercus* wood powder by selected 4 transformants (M1, M8, M12 and M14) displaying the 2 highest levels of MnP activity at 3 and 6 days culture each was carried out. As shown in Table 2, the mass loss of lignin caused by all four p*PbGPD-*MG*mnp2* transformants was greater than that of *HPT* transformants. Transformants M12 and M14 showed a significant increase in lignin mass loss compared with the other transformants; however, there was no difference in the weight loss of *Quercus* wood powder among the transformants. The lignin mass loss to wood powder ratio (*L/W*) of all four p*PbGPD-*MG*mnp2* transformants was higher than that of the *HPT* transformant, indicating a higher selectivity for lignin degradation. The *L/W* ratio also revealed a significantly higher value for the M12 and M14 transformants relative to the *HPT* transformant. Additionally, the MnP activities of the p*PbGPD-*MG*mnp2* transformants on wood powder were higher than those of Wt and the *HPT* transformant (Figure 1C).

**Table 2:** **Mass loss of Klason lignin and** ***Quercus*** **wood powder,** ***L/W*** **ratio in MG-60 transformants**

Strain	Transformed plasmid		Mass loss of Lignin (% original mass)	Mass loss of wood ***Quercus*** powder (% original mass)	***L/W***
	p ***PbGPD-HPT***	p ***PbGPD-*** MG ***mnp2***			
Wt	-	-	31.83 ± 3.16^a^	13.46 ± 2.41^a^	2.43 ± 0.49^b^
HPT	+	-	11.42 ± 2.57^c^	5.59 ± 0.17^b^	2.04 ± 0.68^b^
M1	+	+	13.93 ± 2.07^c^	5.19 ± 0.17^b^	2.70 ± 0.40^b^
M8	+	+	13.85 ± 2.40^c^	5.12 ± 0.30^b^	2.69 ± 0.80^b^
M12	+	+	20.88 ± 2.57^b^	5.35 ± 0.12^b^	3.90 ± 0.49^a^
M14	+	+	25.26 ± 2.02^b^	5.95 ± 0.15^b^	4.24 ± 0.26^a^

Wt indicates the wild type, HPT indicates the hygromycin resistant transformant, and M1, M8, M12, M14 indicate MG*mnp2* regenerated transformants. The tests, using three flasks per strain, were carried out independently. Data means ± SE (n = 3) and values without a common superscript letter are significantly different at *p* <0.05.

However, both the mass losses of wood powder and of lignin were markedly less in the four p*PbGPD-*MG*mnp2* transformants and the *HPT* transformant than in the Wt (Table 2). This may be due to the delayed growth of transformed strains on wood. While there was no difference in the growth rate between Wt and transformed strains on PDA and Kirk media, the growth rate of transformed strains on *Quercus* wood powder medium was markedly slower (Additional file 1: Table S2). Even so, the *L/W* ratio of the M14 transformant was significantly higher than that of Wt. These results suggest that the enhancement of MnP2 activity promotes Klason lignin degradation.

There is a possibility that the decreased ability of transformants to decompose wood was caused by protoplast generation. Many basidiomycete fungi exist as dikaryons in which the two genomes exist in separated nuclei. In previous studies, it has been indicated that the nuclei number was changed during the preparation of protoplasts from dikaryotic mycelia of basidiomycetes (Ohmasa et al. 1987). It has also been reported that the characteristics of progenitor basidiomycetes became altered by protoplast isolation, including growth rate changes (Clark and Anderson 2004). In our study, there was some variation in the sizes of protoplasts prepared from MG-60, and the number of nuclei stained by propidium iodide also varied among the protoplasts (data not shown). It might occur possible that the change in the number of nuclei by protoplast isolation transmuted the original characteristics of MG-60 with regards to delignification; however, it was unclear whether protoplasts of MG-60 were haploid or diploid because the clamp connection was not observed in MG-60.

In summary, our findings demonstrate that the MG-60 strain is able to be co-transformed, and the transformants have MG*mnp2* transgene expression. This MG*mnp2* expression induced an acceleration of lignin degradation, while the original ability to degrade lignin was decreased by protoplast regeneration of the MG-60 strain. Based on these findings, we continue to select protoplast regeneration strains that had a higher ability to degrade lignin than Wt and to analyze whether there is a correlation between the change in delignification and the number of nuclei.

## Electronic supplementary material

Additional file 1: Table S1: Oligonucleotides used as primers in this study. **Table S2.** Mycelial elongation of Phlebia sp. strain MG-60 on PDA or Quercus wood powder media. **Figure S1.** Construction of *HPT, EGFP* and MG*mnp2* expression plasmids. The procedure used to construct p*PbGPD-HPT*, p*PbGPD-EGFP* and p*PbGPD*-MG*mnp2* is described in the text. The horizontal arrows indicate the location and directions of primers. Boxes indicate genes. **Figure S2.** Confirmation of EGFP fluorescence of *EGFP* transformants in *Phlebia* sp. strain MG-60. The EGFP fluorescence of *EGFP* transformants was observed using a BX51 OLYMPUS fluorescence microscope system. (PDF 690 KB)

## Abbreviations

MnP: Manganese peroxidase
*HPT*: Hygromycin- resistance gene
*EGFP*: Enhanced green fluorescent protein gene
GPD: Glyceraldehyde-3-phosphate dehydrogenase.
