# Supplementary material for: Expression of a manganese peroxidase isozyme 2 transgene in the ethanologenic white rot fungus Phlebia sp. strain MG-60
Source: Springerplus. 2014 Nov 27;3:699. doi: 10.1186/2193-1801-3-699 (PMC4447749; doi:10.1186/2193-1801-3-699)
Supplement: Supplementary file 1 — Additional file 1: Table S1: Oligonucleotides used as primers in this study. Table S2. Mycelial elongation of Phlebia sp. strain MG-60 on PDA or Quercus wood powder media. Figure S1. Construction of HPT, EGFP and MGmnp2 expression plasmids. The procedure used to construct pPbGPD-HPT, pPbGPD-EGFP and pPbGPD-MGmnp2 is described in the text. The horizontal arrows indicate the location and directions of primers. Boxes indicate genes. Figure S2. Confirmation of EGFP fluorescence of EGFP transformants in Phlebia sp. strain MG-60. The EGFP fluorescence of EGFP transformants was observed using a BX51 OLYMPUS fluorescence microscope system. (PDF 690 KB) [file 40064_2014_1578_MOESM1_ESM.pdf]

Additional file 1

Table S1. Oligonucleotides used as primers in this study.

Name	Nucleotide sequence (5′- sequence -3′)
PbGPD-F1	GGACGACAGCAGTCGACATA
PbGPD-R1	ATGCTGCGTTCATTGTTCTG
PbGPD-Asc-F1	GCGGCGCGCCCTAAGCGGCTCGTGAGGC
PbGPD-Asc-R1	GGGCGCGCCTTTCAAGTAGGGTGATGTGG
gHPT-Asc-F1	GGGCGCGCCCGGCGGCATGAAAAAGCCT
gHPT-Asc-R1	GGGCGCGCCCTATTCCTTTGCCCTCGGAC
gEGFP-Asc-F1	GGGCGCGCCCATGGGCTCGGGCGGCGGCT
gEGFP-Asc-R1	GGGCGCGCCTTACTTGTACAGCTCGTCCA
gMG*mnp2-Asc*-F1	GGGCGCGCCATGGCCTTCAACTTTGCG
gMG*mnp2-Asc*-R1	GGGCGCGCCTTACGAGTCGCCTTCGC
PbGPD-prom-F1	GCAAATGCGGATCTCCGATT

Table S2. Mycelial elongation of *Phlebia* sp. strain MG-60 on PDA or *Quercus* wood powder media.

Strain	Mycelial elongation (cm)	
	PDA medium[a]	*Quercus* wood powder medium[b]
Wt	6.10	4.02
HPT	6.00	1.77
M1	6.20	1.64
M8	6.00	1.50
M12	6.00	2.32
M14	6.10	2.66

All strain were cultured at 28°C on PDA medium for 3 d and *Quercus* wood powder medium for 8 d.

[a]Mycelial elongation on PDA medium was measured the diameter of the colonies.

[b]Mycelial elongation on *Quercus* wood powder medium was measured the length of vertical extension from inoculation point in test tube.

Figure S1. Construction of *HPT, EGFP* and MG*mnp2* expression plasmids.

The procedure used to construct p*PbGPD-HPT*, p*PbGPD-EGFP* and p*PbGPD*-MG*mnp2* is described in the text. The horizontal arrows indicate the location and directions of primers. Boxes indicate genes.

Figure S2. Confirmation of EGFP fluorescence of *EGFP* transformants in *Phlebia* sp. strain MG-60.

The EGFP fluorescence of *EGFP* transformants was observed using a BX51 OLYMPUS fluorescence microscope system.

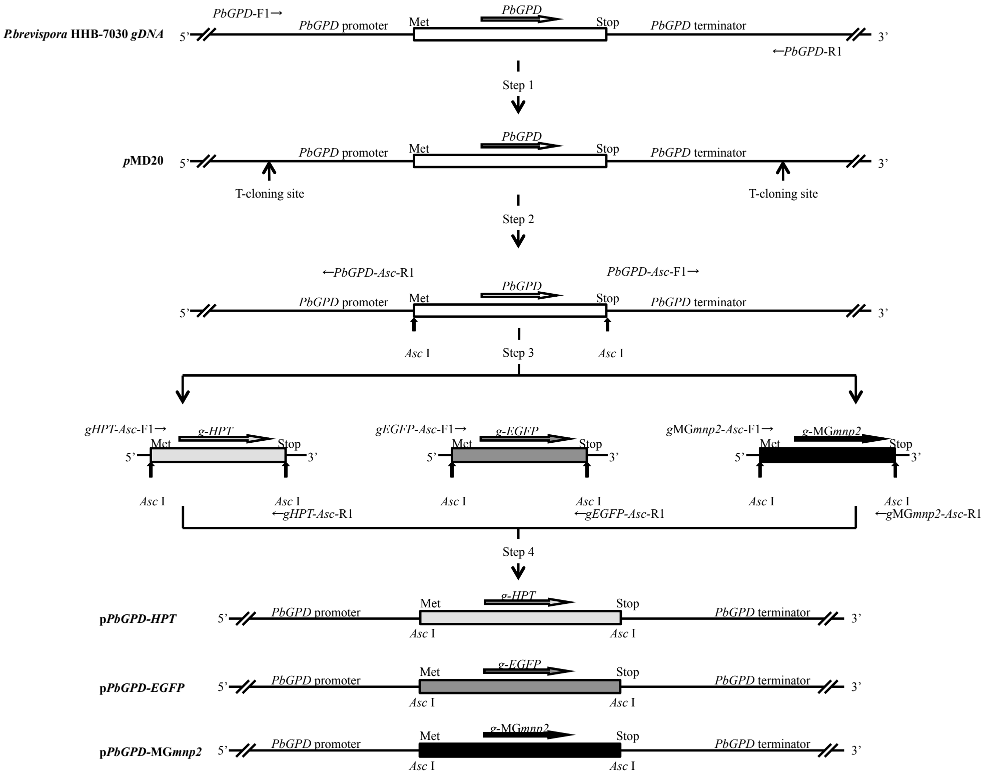

Figure S1. Yamasaki *et al.*

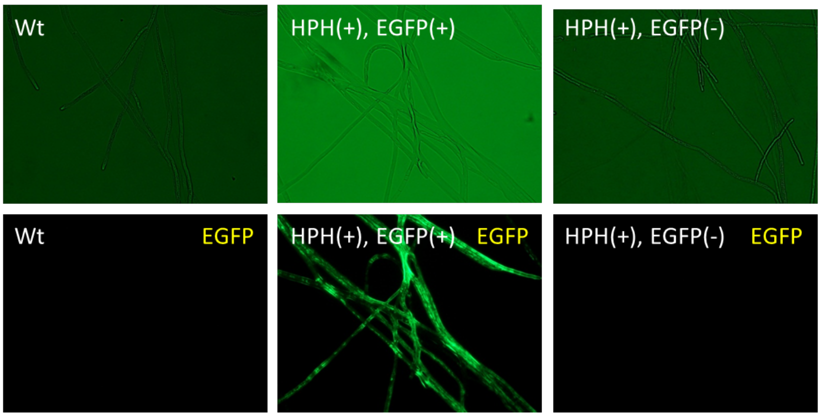

Figure S2. Yamasaki *et al.*
